# Ore image segmentation method using U-Net and Res_Unet convolutional networks

**DOI:** 10.1039/c9ra05877j

**Published:** 2020-03-04

**Authors:** Xiaobo Liu, Yuwei Zhang, Hongdi Jing, Liancheng Wang, Sheng Zhao

**Affiliations:** Intelligent Mine Research Center, Northeastern University Shenyang 110819 China jinghongdi@163.com; National-local Joint Engineering Research Center of High-efficient Exploitation Technology for Refractory Iron Ore Resource, Northeastern University Shenyang 110819 China

## Abstract

Image segmentation has been increasingly used to identify the particle size distribution of crushed ore; however, the adhesion of ore particles and dark areas in the images of blast heaps and conveyor belts usually results in lower segmentation accuracy. To overcome this issue, an image segmentation method UR based on deep learning U-Net and Res_Unet networks is proposed in this study. Gray-scale, median filter and adaptive histogram equalization techniques are used to preprocess the original ore images captured from an open pit mine to reduce noise and extract the target region. U-Net and Res_Unet are utilized to generate ore contour detection and optimization models, and the ore image segmentation result is illustrated by OpenCV. The efficiency and accuracy of the newly proposed UR method is demonstrated and validated by comparing with the existing image segmentation methods.

## Abstract 2

Image segmentation has been increasingly used to identify the particle size of crushed ore. How to accurately identify the ore particles in complex a environment is particularly important.

## Graphical abstract

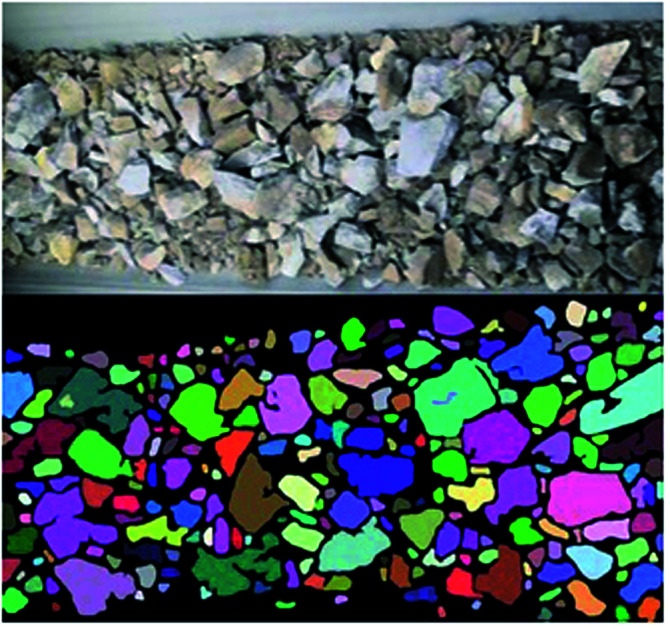

## 1 Introduction

Ore size distribution is a significant indicator for process performance in mining practices. For example, the size distribution of a blast heap is often used to evaluate the blasting effect in stope extraction, which provides the basis for design and optimization of blasting parameters. Additionally, ore size distribution is the key index to evaluate the performance of crushing equipment during mineral processing. In the past decades, ore size detection was finished manually in China, which not only consumed a great amount of labor and material resources but also resulted in low precision and efficiency. With the development of artificial intelligence, automatic detection methods based on advanced image processing technology have been increasingly utilized in ore size detection, in which ore image segmentation (separation of ore particles within one image) is the key step. Therefore, an efficient and accurate method to segment ore images is always of great interest for mining engineers.

Ore image data are mainly extracted from outdoor environments such as blast heaps in stopes and conveyor belts. The ore edge in the image of a blast heap is typically irregular, noisy and fuzzy due to adverse environmental conditions, *e.g.*, huge dust and uneven lighting, in the mining site. The ore in the image of conveyor belt has smaller particle size and more serious adhesion compared with that in the images of blast heaps, which makes ore more difficult to segment. Generally, the image segmentation method can be divided into three categories, *e.g.*, region-, threshold value- and particular theories-based methods. For example, watershed^1–3^ and FogBank^4^ algorithms are often used in region-based segmentation technique; however, they are difficult to segment the ore particles with fuzzy edges, high similarity and adhesion degree accurately. In addition, the region-based method could not handle the ore located in the uneven lighting parts of the image satisfactorily. As for the threshold value based segmentation method, it is one of the most commonly used parallel region techniques and follows certain requirements to determine the gray threshold. For instance, Lu and Zhu^5^ proposed an effective particle segmentation method, in which background difference method and local threshold method were used to eliminate the droplets in crystals and particle shadow, respectively. However, this method is not suitable for ore segmentation with overlapping. Zhang and Liu^6,7^ adopted dual-window Otsu threshold method to determine the threshold value. Although the influence of noise was reduced and the thresholding performance of uneven lighting images was improved efficiently, their method still could not segment the ore images with overlapping and fuzzy boundaries accurately. Particular theories based method incorporates theories into image segmentation technique. For example, Malladi^8^ permuted a watershed transformation approach to generate superpixels efficiently, but this method was not robust enough and time-consuming. Based on multilevel strategy, Yang and Wang^9^ adopted the marker-based region growing method to carry out image segmentation. Mukherjee and Potapovich^10^ utilized regression-based classifier to learn ore shape features. It was found that the segmentation accuracy of ore particle boundary was improved, but the parameter need to be adjusted manually. Zhang and Abbas^11^ combined wavelet transform and fuzzy c-means clustering for particle image segmentation to reduce the noise and separate touching object.

Evidently, past studies into the image segmentation method have proven instrumental in providing insight on the ore size distribution under various scenarios. However, their performances on ore image obtained from conveyor belt or blast heap with mutual adhesion and shadow are still troublesome and not robust. Additional research is therefore required. Deep convolutional network can be a potential solution to be used in image segmentation, as the image segmentation can be regarded as a binary classification problem at the pixel level. One can reduce the computation time and improve the generalization ability of the network significantly by changing the parameters of deep convolutional network.^12^ Yuan and Duan^13^ applied the HED network to realize the segmentation of the ore image of the conveyor belt. It can effectively segment ores of large size, and it further verifies the feasibility of deep learning for image processing of ore. Hence, this study proposes a ore image segmentation technique UR which bases on the combination of U-Net (convolutional neural networks)^14^ and Res_Unet (deep learning framework) model to overcome the low segmentation accuracy, troublesome parameter adjustment and poor adaptability of the existing methods.

## 2 UR-based ore image segmentation method

### 2.1 UR method description

The application of UR method in ore image segmentation includes three stages, *i.e.*, preprocessing, training and testing. The ore image collected from conveyor belt and blast heap at open pit mine usually have bad noise, which requires preprocess to avoid serious over-segmentation. The captures images are firstly manually labeled in Photoshop, and then the labeled images are preprocessed by using gray-scale, median filtering and adaptive histogram equalization techniques. Gray-scale technique aims to transfer the binary image into grayscale, median filtering is used to reduce image noise, and adaptive histogram equalization is utilized to extract ore target region and separate ore targets.

After preprocessing, conveyor belt sample set A (labeled and preprocessed ore images from conveyor belt) and blast heap sample set B (labeled and preprocessed ore images from blasting heap) are trained by U-Net and Res_Unet networks to generate contour detection model A and B respectively. Contour detection models are utilized to verify the sample sets, and then the verification results are binarized to obtain conveyor belt contour atlas (contour set A) and blasting heap contour atlas (contour set B). Later, Res_Unet and U-Net networks are utilized to train contour sets A and B, respectively. The training models with the best performance are saved as contour optimization model A for conveyor belt images and contour optimization model B for blasting heap images.

As for the testing stage, the test images are identified into blast heap images and conveyor belt images based on the different source, and their contour detection model can be used to obtain corresponding contour images. The contour region of the testing image is extracted through the trained contour detection model and then binarized. However, it is worth mentioning that there still exists under-segmentation and small holes in the binarized contour image, which is not convenient for the statistical processing and subsequent operations. Therefore, the ore contours need to be optimized using the trained contour optimization model. Finally, the OpenCV is incorporated to calculate ore size distribution and visualize the segmentation result.

### 2.2 Network structure

#### 2.2.1 U-Net network

**Fig. 1:**
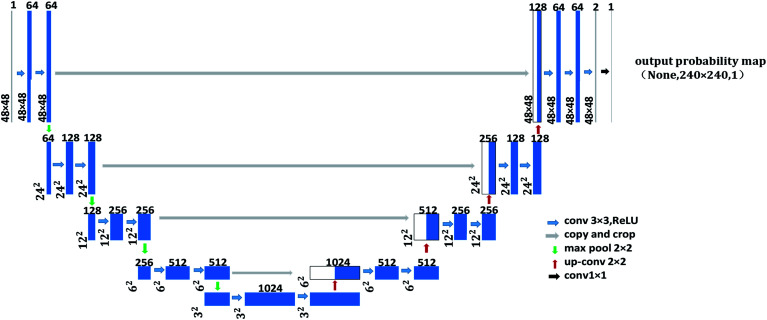
The U-Net network structure of contour detection model A.

U-Net network is a fully convolutional network developed by Long *et al.* in 2017,^15^ which requires fewer training sets and has higher segmentation accuracy compared with other convolutional neural networks. The u-shape structure of U-Net network consists of two parts, *e.g.*, contracting path and expanding path, see Fig. 1. The contracting path is used to get context information, while the expanding path is used for precise positioning.

**Fig. 2:**
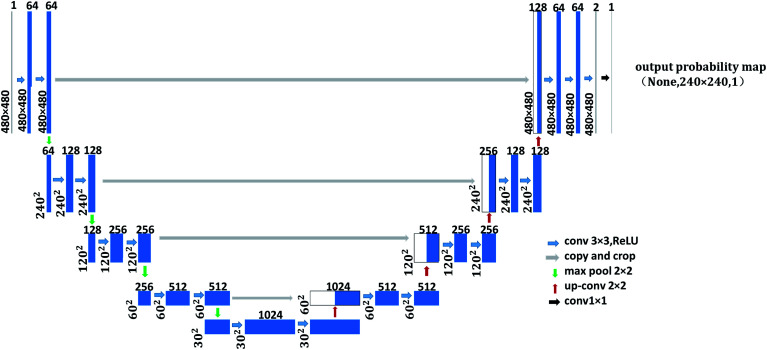
The U-Net network structure of contour optimization model B.

As shown in Fig. 1, the left side is the contracting path that composes of a repeated 3 × 3 convolution kernel and a 2 × 2 maximum pooling layer. ReLu is used in the activation function and the number of characteristic channel would be doubled after each sampling. The right side in the figure is the expanding path, in which the number of characteristic channel is halved by deconvolution in each step. Then, the deconvolution result is spliced with the corresponding contraction path feature graph. Later, the spliced feature graph is convolved twice by 3 × 3. At the last layer of the expanding path, the 1 × 1 convolution kernel is adopted to map each 2-bit eigenvector to the output layer of the network. In this study, contour detection model A and contour optimization model B are both trained by U-Net network with 23 convolution layers. The difference is that input image resolution of contour detection model A is 48 × 48, while that for contour optimization model B is 480 × 480, as shown in Fig. 1 and 2. In the structure figure, the blue box represents multi-channel feature graph, in which image resolution is located at the lower left corner. The white box represents the copied feature graph and the number of channels is indicated at the box top. The network employs sigmoid function and cross entropy as the activation function of neurons and the cost function respectively, which could increase the speed of weight updating and thus improve training speed effectively.

#### 2.2.2 Res_Unet network

**Fig. 3:**
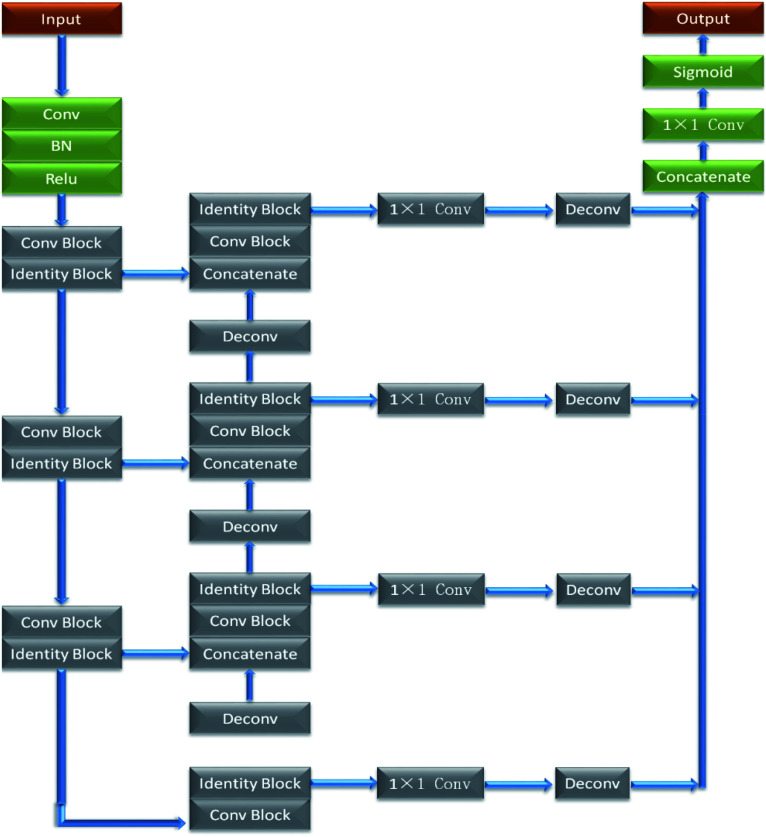
The structure of Res_Unet network.

Res_Unet is a semantic segmentation model based on ResNet (residual neural network)^16^ and U-Net. Res_Unet network integrates residual module and U-Net network capable of effectively overcoming excessive parameters and gradient dispersion caused by the deepened network layer. In addition, new residual learning unit is easy to train in Res_Unet, which not only improves the model training speed, but also enables the network to get fewer parameters without decreasing accuracy. The structure of Res_Unet network is shown in Fig. 3, where both ‘Conv Block’ and ‘Identity Block’ belong to the residual module. ‘BN’ refers to ‘BatchNormalization’, which keeps uniform distribution of the input of each layer for the neural network during the entire training process. ‘Concatenate’ splices the feature map with same size in up- and down-sampling processes to achieve better reconstruction result.

In order to increase the readers' understanding of the network structure in this paper, and to reproduce and further optimize the methods in this paper. Therefore, the specific meaning of some parameters of network structure is expressed in Table 1.

**Table 1:** Network structure description

Number	Name	Meaning	Application
1	Conv 3 × 3	3 × 3 convolution layer	Extract image features
2	Copy and crop	Skip-connection	Splice feature maps
3	Max pool	Maximum pool layer	Dimension reduction
4	Up-conv	Up-sampling process	Restore dimensions
5	Conv 1 × 1	1 × 1 convolution layer	Output results
6	Conv block	Residual module	Amplify the dimension of feature layer
7	Identity block		Change dimensional depth
8	Deconv	Up-sampling process	Increase dimensional information
9	BN	Batch normalization	Data normalization
10	Sigmoid	Activation function	Make network have the ability of layered nonlinear mapping learning
11	ReLu		
12	Concatenate	Connection layer	Connect the last layer of the network to all the previous layers

### 2.3 Contour detection

**Fig. 4:**
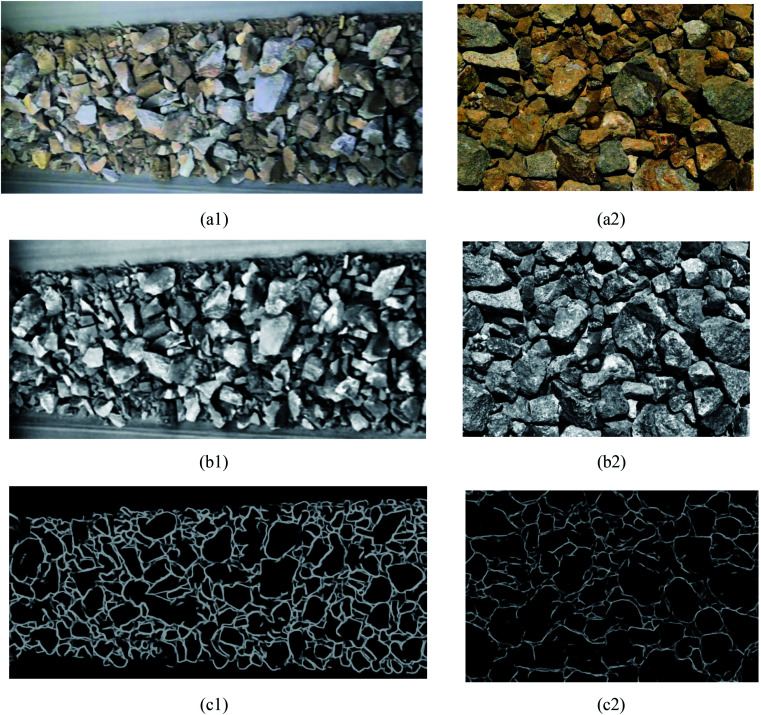
The result of contour detection. (a1) Conveyor belt original image, (a2) original burst image, (b1) the conveyor preprocesses the image, (b2) image preprocessing of blast heap, (c1) belt image contour detection, (c2) blast heap image contour detection.

As discussed earlier, all the ore images require to be preprocessed by using gray-scale, median filter^17^ and adaptive histogram equalization techniques. As shown in Fig. 4(a1 and b2), the image noise is reduced and the gap between ore particles is more evident after image preprocessing. Additionally, this preprocessing method adopts single-channel grayscale graphs as the training set and testing figures, which could reduce the amount of data processing for contour detection model and improve training speed simultaneously. Fig. 4(c1 and c2) are the detected contour for ore particles in conveyor belt and blast heap respectively.

### 2.4 Contour optimization

As shown in Fig. 4, the contour of the ore region has been obtained with the pre-training model, but there are several shortcomings such as boundary discontinuity and over-segmentation. Hence, the contours need to be optimized for the following accurate segmentation. Contour optimization is to obtain a closed and more accurate ore region contour *via* filling holes and completing edges of the contour map.

**Fig. 5:**
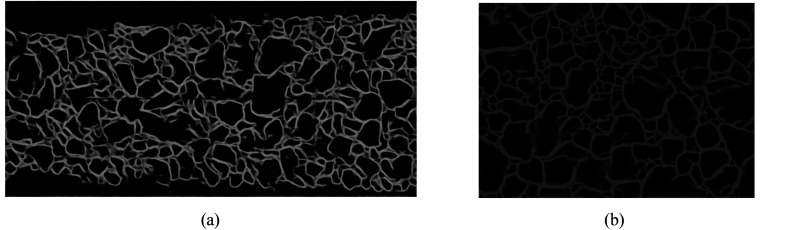
Conveyor belt contour optimization results. (a) Image contour optimization results of conveyor belt. (b) Image contour optimization results of blasting heap.

There are many algorithms to solve the image over-segmentation; however, the parameters calibration involved in is too complex and the result is often not satisfied. Hence, deep learning technique is adopted in this study to realize contour optimization. The trained U-Net and Res_Unet models are used to optimize the ore contour in the primary contour images of conveyor belt and blasting heap respectively as shown in Fig. 5.

### 2.5 Connected region marker

It is worth mentioning that boundary discontinuity and over-segmentation could be solved by using optimization method, but small holes, miscellaneous spots and conveyer belt area disturbance still exist in the contour image. In order to further improve the accuracy of connectivity threshold labeling and increase the detection accuracy of ore particle sizes, it is necessary to mark the connected region in the optimized contour image. In this study, the OpenCV correlation algorithm is employed to realize the connected domain marking for both blasting heap and conveyor belt images.

The threshold value of contour screening condition in OpenCV is set to 0.3 in this study to binarize optimized contour images. The OpenCV correlation algorithm is used to obtain the following parameters of binarization contour images. All contours in contour image are identified *via* ‘findContours’ algorithm. ‘BoundingRect’ algorithm is used to get the minimum bounding rectangle of each contour. ‘ContourArea’ algorithm is utilized to get the area of each contour. ‘ArcLength’ algorithm is employed to get the perimeter “L” of each contour. Moreover, this study adopts different ways to mark connected regions for blasting heap and conveyor belt images.

#### 2.5.1 Connected region marking steps for ore image in blasting heap

The detailed steps for marking connected region in ore contour image of blasting heap is given as followings:

Step 1: Draw probability diagram. After testing image is preprocessed and verified by contour detection model, probability diagram 1 can be obtained. Probability correction diagram *n* − 1 is verified by contour optimization model to obtain probability diagram *n*. Set a parameter *K* and its value for each contour in binarization probability diagram is calculated as:1
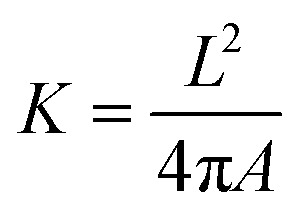
where *L* and *A* are the perimeter and area of the contour respectively.

Step 2: Draw contour diagram. Preset a value *h*, and then employ OpenCV to draw the contours of binary probability diagram *n* that meets *k* < *h* and 
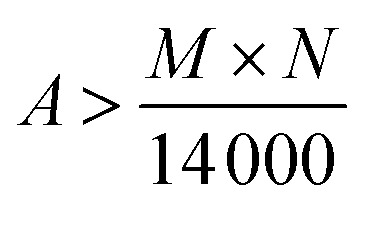
, and get contour diagram *n* and *S*_*n*_ value by2
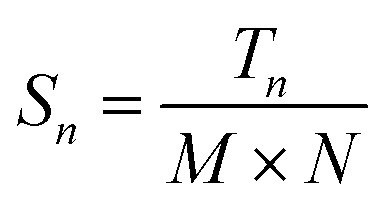
where, *T*_*n*_ is that sum of the areas of all contours that satisfy the drawing conditions in binarization probability graph *n*, and *M* × *N* is that resolution of probability diagram (480 × 480 in this study).

**Fig. 6:**
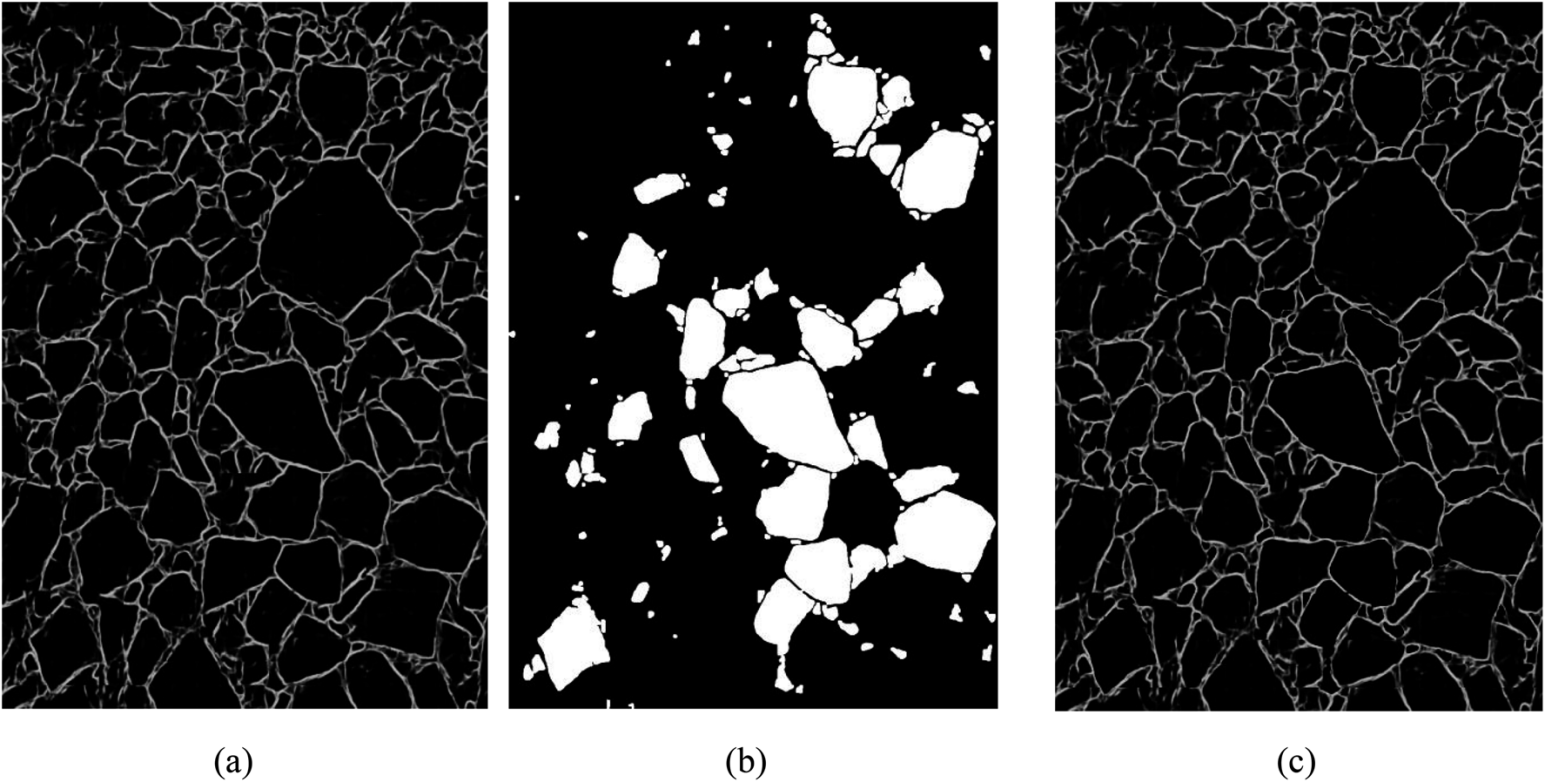
Draw effect of probability correction map. (a) Probability diagram 1, (b) contour diagram 1, (c) probability correction diagram 1.

Step 3: Draw probability correction diagram. OpenCV is used to combine contour diagram *n* with probability diagram 1 to obtain probability correction *n*. The setting of probability correction graph can effectively decrease the miscellaneous points and small holes in probability diagram. Fig. 6 shows the drawing result of probability correction diagram of a test graph.

Step 4: Set *n* = 1, *S*_*n*_ = 0, and *h* = 2. Firstly, through probability diagram, contour diagram *n*, and probability correction diagram *n* are obtained by OpenCV.

Step 5: After testing the probability correction graph *n* with contour optimization model, set *n* = *n* + 1. The probability diagram *n* can be obtained, and then the contour diagram *n* and *S*_*n*_ can be obtained by OpenCV. If *h* ≤ 4 and *S*_*n*−1_ < *S*_*n*_, then go to step 6; if *h* ≤ 4 and *S*_*n*−1_ ≥ *S*_*n*_, then *h* = *h* + 0.5 and go to step 7; otherwise (*h* > 4), go to step 8.

Step 6: Probability correction diagram *n* is obtained by using probability diagram *n* and contour diagram *n*. Then, return to step 5.

Step 7: Set *n* = *n* − 1, and return to step 5.

Step 8: Probability diagram *n* − 1 is verified by contour optimization model to obtain probability diagram *n*. The final contour map is obtained by combining ore regions in binarization probability diagram *n* with the ore regions in contour diagram *n* − 1. Finally, the segmentation result graph is drawn by OpenCV.

#### 2.5.2 Connected region marking method for ore image on conveyor belt

The detailed steps for marking connected region in ore contour image of blasting heap is given as followings:

Step 1: Set initial values of variable *K*, Num, *A*1 and *A*2 as 0, respectively. Since the obtained ore areas in contour map contains small holes, miscellaneous points and interference of conveyor belt areas.3
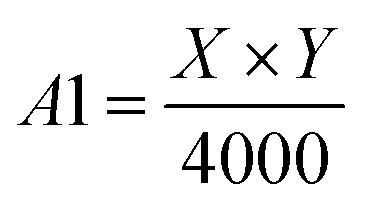
4
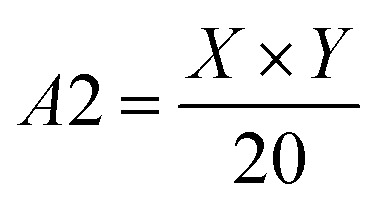
where, *X* × *Y* represents the resolution of the contour images, 48 × 48 in this study.

Step 2: If the contour satisfies *A*1 < *A* < *A*2 and *K* < 6, then let Num = Num + 1. Additionally, the length, width and contour area of minimum external rectangle of the contour is unchanged. Draws all contours that satisfy the above conditions obtain segmentation result graph by drawContours algorithm in OpenCV, and Num is the amount of ore finally detected.

## 3 Experiment

The training of deep convolutional neural networks involved in this study were carried out on GPU and Geforce GTX 1060 6GB under the framework of deep learning Tensorflow and Keras.^18^ OpenCV in python3.6 environment was used to program connected region marking and ore size statistics. Image acquisition device is Huawei nova 3 mobile phone, the rear camera of the device is used to acquire images, and acquired image resolution is 1080 × 1440. Both the ore images on conveyor belt and in blasting heap were photographed from Yanqianshan Iron Open Pit mine located in Liaoning Province, China.

### 3.1 Image data set preparation

#### 3.1.1 Conveyor belt images acquisition

**Fig. 7:**
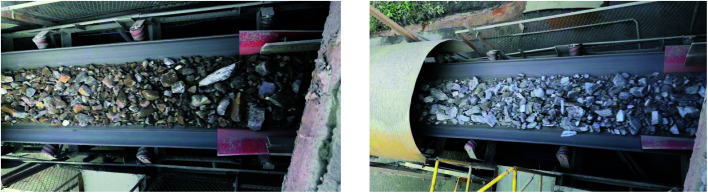
Conveyor belt operation image.

The ore particles have different size from the view of different angles, and ore grade and light conditions also influence the surface color of ore particles. Hence, the collected images or videos should consider all the these factors to ensure and replicate the environmental complexity as shown in Fig. 7.

When collecting conveyor operation video, the vertical distance between camera lens and working surface of conveyor belt is about 3 m, and the horizontal distance is 0–1 m. In order to reduce the complexity of the training set and improve training speed, a total of 39 images that do not contain overlapping regions were selected from collected images. 29 image were used as the training sample set and the remaining 10 images were treated as the testing sample set. The training sample set were firstly preprocessed and then cropped to obtain the images containing the ore region. Later, the interpolation of training sample set images were adjusted to 960 × 480. Finally, the edge lines of ore particles were drawn manually with Photoshop software to make training label set. Both training sample set and it corresponding label set were considered as training set for ore on conveyor belt (training set A). Similarly, the testing sample set and testing label set were selected as testing set for ore on conveyor belt (testing set A).

Training set A was verified by the contour detection model A to obtain binarized contour set, and the binarized contour set and its corresponding label set were combined into the training set 2. The total number of training samples in contour detection model A was 725 000, which were randomly selected from 29 images in training set A. 25 000 samples with a resolution of 48 × 48 were taken from each image on average. The total number of training samples in contour optimization model A was 29 000, which were randomly selected from 29 images in training set 2. On average, 1000 samples with a resolution of 240 × 240 were taken from each image.

#### 3.1.2 Blast heap images acquisition

In order to improve the practical application of the method, the linear distance between camera lens and the surface of explosion pile is 1–8 m. 27 blasting heap images were collected from the open pit mine, in which 17 images were chosen as training sample set and the remaining images were selected as testing sample sets. In order to create a deep learning sample set that meets the requirement of experimental comparison, the images in the training sample set after preprocessing are cropped to obtain the images containing the ore region. The edge lines of ore particles were manually drawn by Photoshop as the label set. Training sample set and its corresponding label set are combined into training set B, while testing sample set and its corresponding label set were taken as testing set B.

The total number of training samples for contour detection model B was 850 000, which were randomly selected from 17 images in training set B. Each sample for contour detection model B had a resolution of 48 × 48. The total number of training samples for contour optimization model B is 11 900, which were randomly sampled from 17 probability diagrams. The probability diagrams were obtained by testing sample set with contour detection model B. The resolution of sample from the probability diagram was 480 × 480.

### 3.2 Model training

In this study, both U-Net and Res_Unet networks were used to in the training process of conveyor belt and blasting heap images, but the training parameters were different as given in Tables 2 and 3 respectively. In the table, ‘Batch_size’ represents the number of images per iteration; ‘Epochs’ means the number of computations for all sample data; ‘Imgs_train’ represents the total number of training sample; and ‘Resolution’ refers to the resolution of training sample. Model training used stochastic gradient descent method.

**Table 2:** Conveyor belt image training parameters

Training model	Contour detection model A	Contour optimization model A
Resolution	48 × 48	240 × 240
Imgs_train	725 000	29 000
Batch_szie	1	1
Epochs	5	7

**Table 3:** Blasting heap image training parameters

Training model	Contour detection model B	Contour optimization model B
Resolution	48 × 48	480 × 480
Imgs_train	850 000	11 900
Batch_szie	1	1
Epochs	5	10

Contour detection model A was trained by U-Net network with input resolution of 48 × 48, and the entire training cost about 85 hours. Res_Unet network with input resolution of 240 × 240 was used for training contour optimization model A, which took about 9 hours. As for the contour detection B and contour optimization B, they were trained by Res_Unet (65 hours) with input resolution of 48 × 48 and U-Net (20 hours) with input resolution of 480 × 480 respectively.

### 3.3 Performance indicators

To evaluate the performance of the proposed image segmentation algorithm, a mathematical model is established. The image segmentation result graph and manual stroke graph are binarized respectively. The inner pixel of each contour in the image is determined as 1, while other pixels are set for 0. The segmentation binary graph and manual stroke graph are selected as algorithm diagram and standard diagram respectively. In this study, three performance indicators, *i.e.*, segmentation precision (SA), over-segmentation rate (OS) and under-segmentation rate (US) are used to evaluate the segmentation algorithm:5
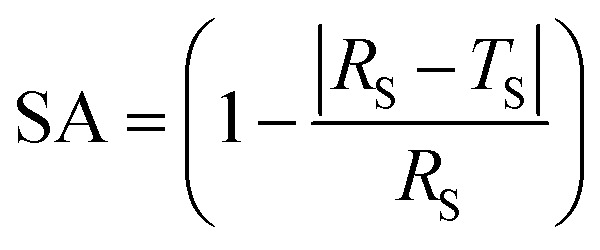
6
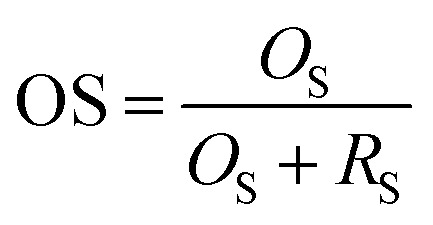
7
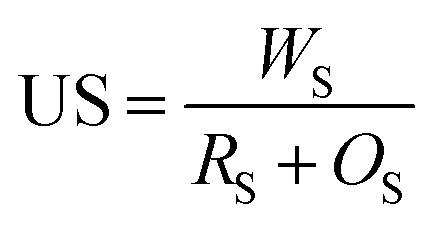
where *R*_S_ is the number of pixel points in the standard diagram with pixel value of 1; *T*_S_ the number of pixel points with pixel values of 1 in both algorithm and standard diagrams; *O*_S_ the number of pixel points with pixel value of 1 and 0 in the algorithm and standard diagrams respectively; and *W*_S_ the number of pixels with pixel value of 0 and 1 in the algorithm and standard diagrams respectively.

## 4 Results and discussions

**Fig. 8:**
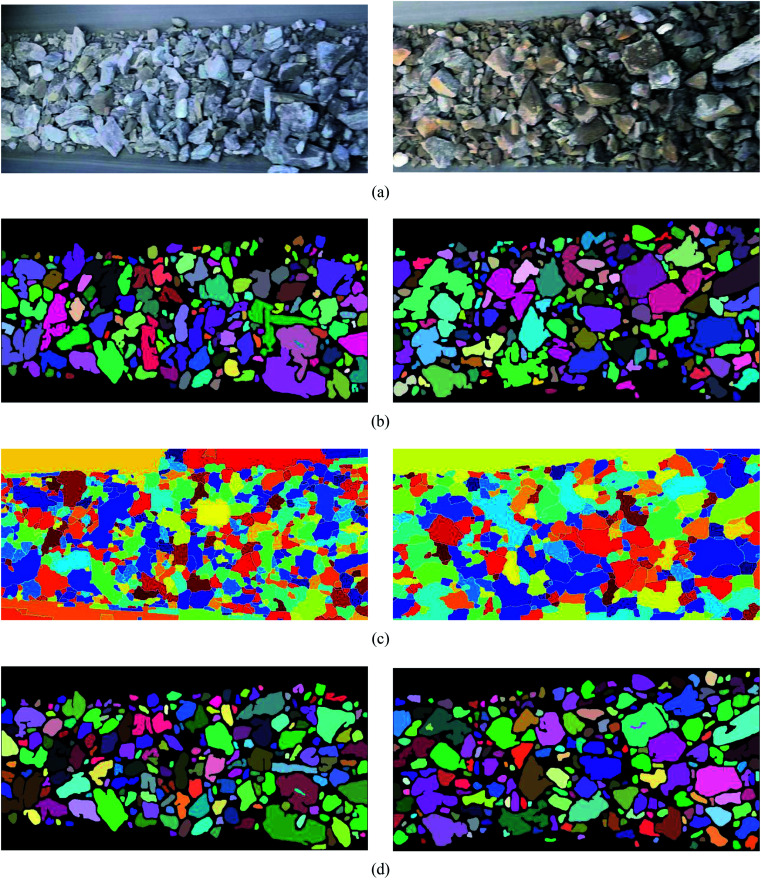
The segmentation results of the conveyor images are obtained by different methods. (a) Original image, (b) NUR method, (c) watershed algorithm based on morphological reconstruction, (d) UR method.

**Fig. 9:**
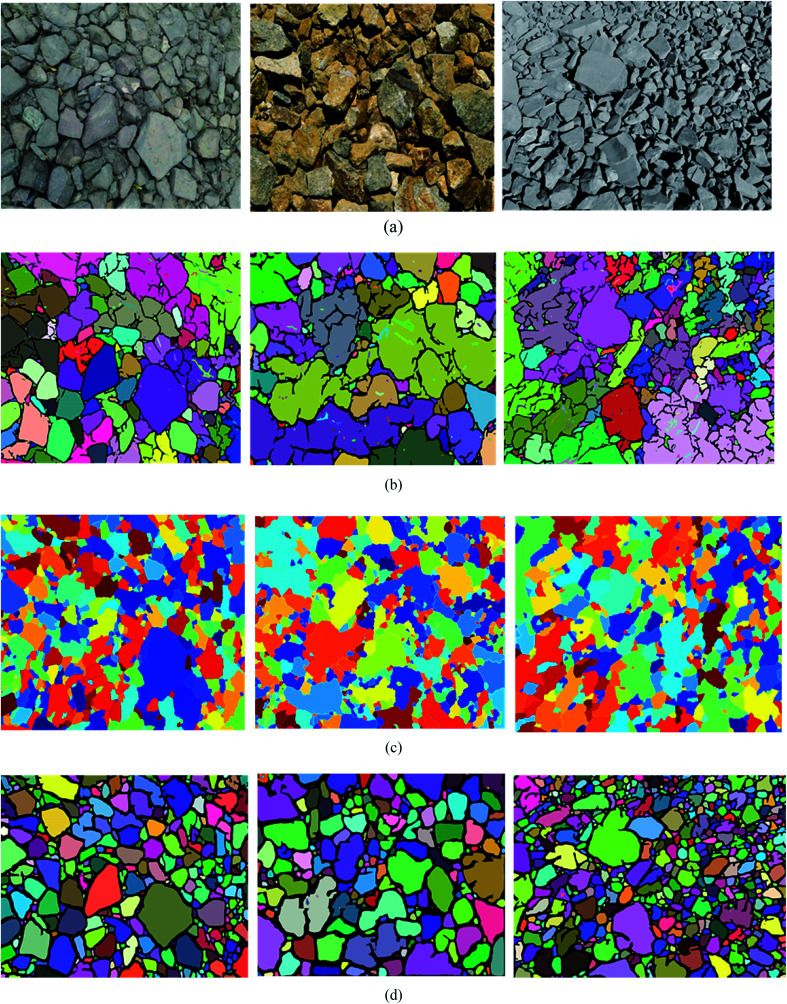
Different methods are used to segment the results of blast heap images. (a) Original image, (b) NUR method, (c) watershed algorithm based on morphological reconstruction, (d) UR method.

To demonstrate the superiority of UR method in ore image segmentation, watershed method based on morphological reconstruction^19^ and NUR method (UR method without Res_Unet contour optimization) are also used to train and test the ore images obtained from open pit mine. The performance indicators of these methods for each testing image is listed in Tables 4 and 5. The segmentation results of these three methods for ore image on testing set A (conveyor belt) with different photographing angles and ore colors are compared in Fig. 8, while that on testing set B (blasting heap) with different ore particle numbers and ore colors are illustrated in Fig. 9.

**Table 4:** The performance index of the test set of conveyor belt is divided by different methods

Method	Performance indicators	Image 1	Image 2	Image 3	Image 4	Image 5	Image 6	Image 7	Image 8	Image 9	Image 10	Mean
NUR method	SA	0.9801	0.8145	0.8432	0.8271	0.9613	0.9676	0.9444	0.9398	0.9169	0.8844	0.9079
	US	0.1095	0.2176	0.1849	0.1975	0.1177	0.1211	0.15	0.1553	0.1594	0.1752	0.1588
	OS	0.0915	0.0395	0.0334	0.0297	0.0821	0.0916	0.1	0.1013	0.0832	0.0673	0.072
Watershed algorithm	SA	0.6935	0.7323	0.7233	0.7702	0.611	0.5602	0.447	0.5778	0.6396	0.685	0.644
	US	0.1027	0.1219	0.1142	0.1584	0.1035	0.1089	0.0927	0.1155	0.1172	0.1259	0.1161
	OS	0.3132	0.3073	0.3062	0.3157	0.3546	0.3811	0.4158	0.3781	0.351	0.3353	0.3458
UR method	SA	0.9309	0.9197	0.9105	0.9625	0.9394	0.9382	0.942	0.9106	0.9981	0.951	0.9403
	US	0.069	0.0627	0.1333	0.099	0.0649	0.0696	0.0848	0.0622	0.1106	0.1377	0.0894
	OS	0.1292	0.1324	0.0481	0.0639	0.1183	0.1238	0.135	0.1392	0.1123	0.0923	0.1095

**Table 5:** The performance index of the test set of blasting heap is divided by different methods

Method	Performance indicators	Image 1	Image 2	Image 4	Image 5	Image 6	Image 7	Image 8	Image 9	Image 10	Mean
NUR method	SA	0.7744	0.8124	0.9195	0.8956	0.8604	0.9749	0.9313	0.9089	0.8804	0.8845
	US	0.0385	0.0325	0.0424	0.0686	0.0256	0.0746	0.0613	0.0503	0.0422	0.0466
	OS	0.2155	0.1854	0.1138	0.1567	0.1449	0.0973	0.1216	0.1296	0.1446	0.1438
Watershed algorithm	SA	0.7676	0.8923	0.9383	0.8949	0.9072	0.9807	0.8758	0.8358	0.929	0.8882
	US	0.1039	0.116	0.1072	0.1027	0.1017	0.129	0.0851	0.069	0.1372	0.1005
	OS	0.2729	0.202	0.1591	0.188	0.178	0.1455	0.1862	0.2003	0.1944	0.1896
UR method	SA	0.8174	0.9811	0.943	0.9393	0.9359	0.9774	0.9539	0.9127	0.8799	0.9265
	US	0.0785	0.1463	0.1354	0.0891	0.0704	0.1238	0.0899	0.069	0.0595	0.1005
	OS	0.2208	0.1298	0.0832	0.1412	0.1264	0.1035	0.13	0.1438	0.1604	0.1313

It is evident from Table 4 that, for ore images on conveyor belt UR method has a higher segmentation accuracy with mean SA value of 0.9403 compared with watershed method (0.6440) and NUR method (0.9079). In addition, the under-segmentation rate of UR method (0.1095) is clear lower than that of watershed (0.3458) and NUR (0.1588) methods. However, it is worth mentioning that UR method has a higher over-segmentation rate than NUR method. Similar results can be noticed when these three methods are utilized in ore images obtained from blasting heap, see Table 5. The image segmentation results is also illustrated in Fig. 8 and 9. As seen, the watershed algorithm is not suitable for extracting ore particle edges in both conveyor belt and blasting heap conditions as shown in Figs. 8(c) and 9(c), and the influence of conveyor belt could not be excluded. It is because watershed method requires constant debugging of the size of structural elements for open and closed reconstruction. As for NUR method, even though it extracts the ore contour more accurately, the significant over-segmentation is observed. UR method provides satisfied segmentation results for ore image obtained from both conveyor belt and blasting heap. Additionally, the adjustment of parameters is not required in UR method, which is more convenient and efficient compared with watershed and NUR methods.

## 5 Conclusion

A new image segmentation method UR for ore particles on open pit mine (conveyor belt and blasting heap) is proposed in this study, which aims to deal with the ore image with uneven size distribution, fuzzy edges and mutually adhesion. The UR method is a deep learning network with the combination of U-Net and Res_Unet models, which includes three stages including preprocessing, training and testing. Gray-scale, median filtering and adaptive histogram equalization techniques are used to preprocess the ore images captured from mine site, and OpenCV is used to illustrate the segmentation results. It is demonstrated that the UR method could exclude the influence of conveyor belt region and avoid complex parameter adjustment. Compared with the existing ore image segmentation method such as watershed and NUR methods, UR method performs higher segmentation accuracy and lower over-segmentation rate.

## Conflicts of interest

There are no conflicts to declare.

## Supplementary Material

## References

[cit1] Wang R.-x. , Zhang W.-c. and Shao L.-z., Research of Ore Particle Size Detection Based on Image Processing, Proceedings of 2017 Chinese Intelligent Systems Conference, 2017, vol. 460, pp. 505–514

[cit2] Amankwah A. and Aldrich C., Automatic ore image segmentation using mean shift and watershed transform, Proceedings of 21st International Conference Radioelektronika 2011, 2011, pp. 1–4

[cit3] Done K. and Jiang D.-l., Automated Estimation of Ore Size Distributions Based on Machine Vision, Lecture Notes in Electrical Engineering, 2014, vol. 238, pp. 1125–1131

[cit4] Chalfoun J., Majurski M., Dima A. (2014). *et al.*, FogBank: a single cell segmentation across multiple cell lines and image modalities. BMC Bioinf..

[cit5] Lu Z. M., Zhu F. C., Gao X. Y., Gao X. Y. (2018). *et al.*, In-situ particle segmentation approach based on average background modeling and graph-cut for the monitoring of, l-glutamic acid crystallization. Chemom. Intell. Lab. Syst..

[cit6] Zhang G.-y., Liu G.-z., Hong Z. (2011). Segmentation algorithm of complex ore images based on templates transformation and reconstruction. Int. J. Miner., Metall. Mater..

[cit7] Zhang G. Y., Liu G. Z., Zhu H. (2010). *et al.*, Ore image thresholding using bi-neighbourhood Otsu's approach. Electron. Lett..

[cit8] Malladi S. R. S. P. , Ram S. and Rodriguez J. J., Superpixels using morphology for rock image segmentation, 2014 Southwest Symposium on Image Analysis and Interpretation, 2014, pp. 145–148

[cit9] Yang G.-q., Wang H.-g., Xu W.-l. (2014). *et al.*, Ore particle image region segmentation based on multilevel strategy. Chin. J. Anal. Lab..

[cit10] Mukherjee D. P., Potapovich Y., Levner I. (2009). *et al.*, Ore image segmentation by learning image and shape features. Pattern Recogn. Lett..

[cit11] Zhang B., Abbas A., Romagnoli J. A. (2011). Multi-resolution fuzzy clustering approach for image-based particle characterization for particle systems. Chemom. Intell. Lab. Syst..

[cit12] Duan J., Liu X., Wu X. (2019). *et al.*, Detection and segmentation of iron ore green pellets in images using lightweight U-net deep learning network. Neural Computing and Applications.

[cit13] Yuan L. and Duan Y.-y., A Method of Ore Image Segmentation Based on Deep Learning, Lecture Notes in Computer Science, 2018, vol. 10956, pp. 508–519

[cit14] Ronneberger O. , Fischer P. and Brox T., U-Net: Convolutional Networks for Biomedical Image Segmentation, International Conference on Medical Image Computing and Computer-Assisted Intervention, 2015, vol. 9351, pp. 234–241

[cit15] Shelhamer E., Long J., Darrell T. (2017). Fully Convolutional Networks for Semantic Segmentation. IEEE Trans. Pattern Anal. Mach. Intell..

[cit16] He K.-m. , Zhang X.-y. and Ren S.-q., *et al.*, Deep Residual Learning for Image Recognition, Proceedings of the IEEE Conference on Computer Vision and Pattern Recognition, 2016, pp. 770–778

[cit17] Rui H. , Liu P. and Jia K., An Improved Adaptive Median Filter Algorithm and Its Application, Advances in Intelligent Information Hiding and Multimedia Signal Processing, 2017, pp. 179–186

[cit18] Erickson B. J. (2017). *et al.*, Toolkits and Libraries for Deep Learning. J. Digit. Imag..

[cit19] Wu Y. (2017). *et al.*, Improved image segmentation method based on morphological reconstruction. Multimed. Tool. Appl..

